# Fahr Syndrome Unknown Complication: Overactive Bladder

**DOI:** 10.1155/2014/939268

**Published:** 2014-07-16

**Authors:** Devrim Tuglu, Ercan Yuvanç, Fatih Bal, Yakup Türkel, Ersel Dağ, Erdal Yılmaz, Ertan Batislam

**Affiliations:** ^1^Department of Urology, Faculty of Medicine, University of Kirikkale, Yahsihan, 71100 Kirikkale, Turkey; ^2^Department of Neurology, Faculty of Medicine, University of Kirikkale, Turkey

## Abstract

A 38-year-old male patient was admitted to our outpatient department because of frequency and urgency incontinence. During evaluation it was detected that the patient was suffering from frequency which was progressive for one year, feeling of incontinence, and urgency incontinence. There was no urologic pathology detected in patient's medical and family history. Neurologic consultation was requested due to his history of boredom, reluctance to do business, balance disorders, and recession for about 3 years. Brain computerized tomography (CT) scan revealed that amorphous calcifications were detected in the bilaterally centrum semiovale, basal ganglia, capsula interna, thalami, mesencephalon, pons and bulbus, and the bilateral cerebellar hemispheres. We have detected spontaneous neurogenic detrusor overactivity without sphincter dyssynergia after evaluating the voiding diary, cystometry, and pressure flow study. We consider the detrusor overactivity which occurred one year after the start of the neurological symptoms as the suprapontine inhibition and damage in the axonal pathways in the Fahr syndrome.

## 1. Introduction

Fahr's syndrome, also referred to as idiopathic basal ganglia calcification (IBGC) or bilateral striopallidodentate calcinosis, is a disease characterized by symmetric, nonatherosclerotic, bilateral vascular calcification of the basal ganglia [1]. Clinical symptoms of IBGC are found in the literature as case reports, because the disease is very rare. Clinically, the most common presentation of IBGC is considered to be Parkinsonism or other hyperkinetic movement disorders (chorea, tremor, dystonia, athetosis, orofacial dyskinesia). The second most common presentation of IBGC is cognitive impairment followed by cerebellar impairment and speech disorder. Psychiatric features, gait disorders, sensory changes, and pain are also reported [2].

In our case, we purpose to report “neurogenic detrusor overactivity,” which is an unknown symptom of Fahr's syndrome.

## 2. Case Report

A 38-year-old male patient with a history of boredom, reluctance to do business, balance disorders, and recession for about 3 years was admitted to our outpatient department because of frequency and urgency incontinence.

During evaluation it was detected that the patient was suffering from frequency which was progressive since one year, feeling of incontinence and urgency incontinence. There was no urologic pathology detected in patient's medical and family history. Urine analysis, urine culture, blood urea nitrogen, and creatinine levels revealed normal values. A kidney, ureter, and bladder (KUB) X-ray study is nonpathological. Urinary ultrasound revealed that bilaterally kidneys and urinary bladder had normal values. Uroflowmetry result revealed that volume voided was 224 mL, Qmax was 22 mL/s, Qave was 9 mL/s, and postvoiding volume was 15 mL. We have detected spontaneous neurogenic detrusor overactivity without sphincter dyssynergia after evaluating the voiding diary, cystometry, and pressure flow study. Also neurologic consultation was requested due to his history of boredom, reluctance to do business, balance disorders, and recession for about 3 years. In the neurologic evaluation, the patient was well oriented and cooperative. He was appearing apathetic. The minimental test score was 29.

His finger to nose and rapid alternating movement test in the bilateral upper extremities and his tandem walking test were pathologic. There was no metabolic failure detected in the routine blood tests and hormonal parameters in our case. No pathology was detected in the patient's and his family history. There was no familial sign detected after performing family scanning in our case.

**Figure 1:**
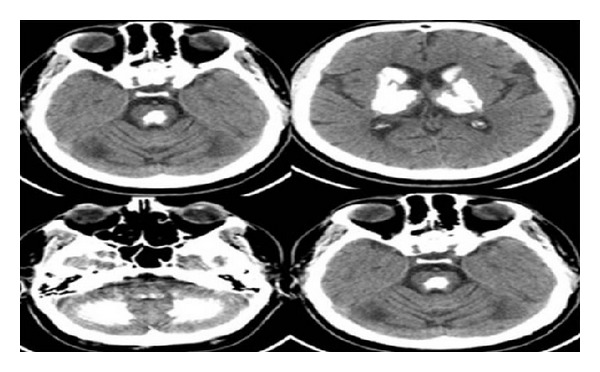
Brain computerized tomography (CT) scan: amorphous calcifications were detected in the bilaterally centrum semiovale, basal ganglia, capsula interna, thalami, mesencephalon, pons and bulbus, and the bilateral cerebellar hemispheres.

Brain computerized tomography (CT) scan revealed that amorphous calcifications were detected in the bilaterally centrum semiovale, basal ganglia, capsula interna, thalami, mesencephalon, pons and bulbus, and the bilateral cerebellar hemispheres (Figure 1).

## 3. Discussion

In 1930, the German neuropathologist Karl Theodor Fahr presented a 55-year-old patient with a history of dementia and hypothyroidism, immobility without paralysis, and the calcifications of the basal ganglia named as Fahr's disease [2, 3].

In 1986, Lowenthal established the defining criteria to the Fahr syndrome: (1) calcifications should have a characteristic distribution or inquire at least globus pallidus, with or without cerebellar calcification; (2) the calcifications should be obvious on the computed tomography; (3) the calcifications should be large enough to be detected at macroscopic examination [4].

Also in our case the CT scan revealed lesions like amorphous calcifications in bilateral centrum semiovale, basal ganglia, capsula interna, thalami, mesencephalon, pons bulbus, and the bilateral cerebellar hemispheres.

There was no metabolic failure detected in the routine blood tests and hormonal parameters.

Another point to mention in differential diagnosis is juvenile Parkinsonism. Although urodynamic findings in juvenile Parkinsonism reveal neuropathic detrusor activity, the cranial computed tomography findings are normal. But in our case pathognomonic findings of Fahr syndrome were observed in cranial computed tomography in addition to urodynamic findings [5, 6].

The voiding dysfunction is most often characterized symptomatically by frequency, urgency, and urge incontinence and urodynamically by normal sensation with involuntary contraction at low filling volumes. Overactive detrusor function indicates the presence of involuntary detrusor contractions during the filling phase [7].

## 4. Conclusion

We consider the detrusor overactivity which occurred one year after the start of the neurological symptoms as the suprapontine inhibition and damage in the axonal pathways in the Fahr syndrome. After reviewing the current literature about Fahr syndrome, we have not detected any symptoms associated to voiding. With this case we think that Fahr syndrome should be considered in the differential diagnosis by cases with detrusor overactivity accompanying neuropsychiatric or neurodegenerative symptoms.
